# US Veterinarians' Knowledge, Experience, and Perception Regarding the Use of Cannabidiol for Canine Medical Conditions

**DOI:** 10.3389/fvets.2018.00338

**Published:** 2019-01-10

**Authors:** Lori Kogan, Regina Schoenfeld-Tacher, Peter Hellyer, Mark Rishniw

**Affiliations:** ^1^Department of Clinical Sciences, College of Veterinary Medicine and Biomedical Science, Colorado State University, Fort Collins, CO, United States; ^2^Department of Molecular and Biomedical Sciences, College of Veterinary Medicine, North Carolina State University, Raleigh, NC, United States; ^3^Veterinary Information Network, Davis, CA, United States

**Keywords:** cannabidiol, canine, legal status, veterinary use, client communication

## Abstract

Due to the myriad of laws concerning cannabis, there is little empirical research regarding the veterinary use of cannabidiol (CBD). This study used the Veterinary Information Network (VIN) to gauge US veterinarians' knowledge level, views and experiences related to the use of *cannabinoids* in the medical treatment of dogs. Participants (*n* = 2130) completed an anonymous, online survey. Results were analyzed based on legal status of recreational marijuana in the participants' state of practice, and year of graduation from veterinary school. Participants felt comfortable in their knowledge of the differences between Δ9-tetrahydrocannabinol (THC) and marijuana, as well as the toxic effects of marijuana in dogs. Most veterinarians (61.5%) felt comfortable discussing the use of CBD with their colleagues, but only 45.5% felt comfortable discussing this topic with clients. No differences were found based on state of practice, but recent graduates were less comfortable discussing the topic. Veterinarians and clients in states with legalized recreational marijuana were more likely to talk about the use of CBD products to treat canine ailments than those in other states. Overall, CBD was most frequently discussed as a potential treatment for pain management, anxiety and seizures. Veterinarians practicing in states with legalized recreational marijuana were more likely to advise their clients and recommend the use of CBD, while there was no difference in the likelihood of prescribing CBD products. Recent veterinary graduates were less likely to recommend or prescribe CBD. The most commonly used CBD formulations were oil/extract and edibles. These were most helpful in providing analgesia for chronic and acute pain, relieving anxiety and decreasing seizure frequency/severity. The most commonly reported side-effect was sedation. Participants felt their state veterinary associations and veterinary boards did not provide sufficient guidance for them to practice within applicable laws. Recent graduates and those practicing in states with legalized recreational marijuana were more likely to agree that research regarding the use of CBD in dogs is needed. These same groups also felt that marijuana and CBD should not remain classified as Schedule I drugs. Most participants agreed that both marijuana and CBD products offer benefits for humans and expressed support for use of CBD products for animals.

## Introduction

Cannabis is one of the earliest cultivated crops, grown in Taiwan for fiber starting about 10,000 years ago (1). The Emperor Shen-Nung, a pharmacologist, wrote a book on treatment methods in 2737 BCE that included the medical benefits of cannabis and recommended it for many ailments, including constipation, gout, rheumatism, and absent-mindedness (2). Cannabis plants can be genetically classified as either hemp or marijuana, based on the concentration of (-)-Δ9-tetrahydrocannabinol (THC), and other cannabinoids they contain (3). Marijuana typically refers to plants with high concentrations of THC, the psychotropic drug used for medicinal or recreational purposes. In contrast, hemp is typically cultivated for use in personal care products, nutritional supplements, and fabrics. It contains higher amounts of CBD, which does not have psychotropic properties. The rules and regulations for CBD and marijuana are different with each having separate statutory definitions.

Recently, the US senate debated the legalization of industrial hemp, with the introduction of the Hemp Farming Act of 2018, aimed at lifting the ban on hemp as an agricultural commodity. Incorporated into the larger 2018 Farm Bill the hemp farming act was passed. The Hemp Farming Act provides for the removal of industrial hemp from Schedule I of the Controlled Substance Act (CSA). This removal would explicitly legalize the cultivation, processing and sale of all hemp-derived products, including CBD (4). The final stages of this legalization process are yet to develop. In September, 2018 the U.S. Department of Justice and the Drug Enforcement Agency announced that Epidiolex (newly approved CBD containing anti-seizure medication) was placed in Schedule V. The DEA signaled that this approval only applied to Epidiolex and not all CBD products (5).

As such, the legal status of CBD remains confusing. According to the Drug Enforcement Agency, Schedule V drugs, substances, or chemicals are defined as drugs with lower potential for abuse than Schedule IV and consist of preparations containing limited quantities of certain narcotics. Schedule V drugs are generally used for antidiarrheal, antitussive, and analgesic purposes (6).

The confusion around legal status of cannabis has made it challenging to study its effects, yet the demand for recreational and medical cannabis continues to grow. Sales of legal recreational and medical cannabis in the United States in 2017 resulted in $5.8–$6.6 billion revenue, and by 2022, legal cannabis revenue in the U.S. market is projected to reach $23.4 billion (7).

Against this backdrop, research remains minimal. Those wishing to study the effects of cannabis or cannabinoids must navigate a challenging process that may involve the National Institute on Drug Abuse, Food and Drug Administration, Drug Enforcement Administration, offices or departments in their state's government, state boards, their home institution, and potential funders (8). There have been a handful of controlled clinical trials conducted with cannabinoids, reporting positive effects on pain, nausea, vomiting, inflammation, cancer, asthma, glaucoma, spinal cord injury, epilepsy, hypertension, multiple sclerosis, Parkinson's disease, Alzheimer's disease, or loss of appetite (9–11). In late June 2018, the FDA approved Epidiolex, the nation's first drug derived from marijuana, for the treatment of seizures associated with two rare and severe forms of epilepsy in humans (12).

Research on animals is equally challenging, with few researchers studying cannabis in animal patients without explicit FDA and DEA approval, but in a manner they contend complies with federal and state law. A researcher from Colorado State University recently reported findings from a small pilot study involving 16 dogs. She found that 89 percent of epileptic dogs had fewer seizures when taking the chicken-flavored CBD oil, as compared to about 20 percent that had were on a placebo (13). Another project, conducted at Cornell University, included a randomized, placebo-controlled, double-blind crossover study that appeared to show that dogs treated with CBD oil have a clinically significant reduction in pain and an increase in activity (14). Given its growing popularity, it is important to assess small animal veterinary practitioners' experiences with CBD products for dogs. This current study was designed to gauge US veterinarians' knowledge level, views and experiences related to use of *cannabinoids* in the medical treatment of dogs. This study was not designed to study perceptions, views, or experiences related to the use of marijuana products with high levels of THC in dogs. The authors' perception is that there is much more interest in the public for using CBD products in dogs, possibly due to concerns over THC toxicity.

## Method

An anonymous online survey was created, in collaboration with VIN (Veterinary Information Network–an online veterinary community), to evaluate veterinarians' views regarding marijuana and CBD/hemp products. The survey was created and tested for usability by researchers at Colorado State University. After the survey was created, one of the authors of this paper (MR) set up online distribution and arranged for a small sample of VIN members to pilot test the survey for appropriate branching and question flow, ambiguity, and potentially missing or inappropriate response options. Their feedback was analyzed, and incorporated into the final version of the survey. A link to the survey was distributed via an email invitation to all VIN members (*n*~34,000), and access was made available from April 27, 2018- May 16, 2018. A follow-up message was sent 2 weeks after the initial invitation. Only data from respondents who stated they currently treat dogs in clinical practice were included in the study. The study was categorized as exempt by Colorado State University's Institutional Review Board. Because this was an anonymous survey, written informed consent was not required. An introductory statement explained the study and indicated to potential participants that consent was implied by completing the survey.

The survey was administered directly via the VIN data collection portal, and branching logic was used to display only questions relevant to each participant. The first question was a screening tool to ensure respondents were clinical veterinarians practicing in the US. Veterinarians who self-identified as not in a US clinical practice (*n* = 26) or did not treat dogs (*n* = 52) were eliminated from further analysis. The body of the survey consisted primarily of short questions, for which participants were able to select one or more specific options to represent their experiences and perceptions regarding hemp/CBD products. Free-text boxes were provided for participants to enter brief alternative answers when none of the listed options applied to them. A final question at the end of the survey allowed for free-text entry of any comments participants chose to make about hemp/CBD products.

## Results

A total of 2,208 responses were received, 78 of which were eliminated as per above, leaving a sample size of 2,130. Not all survey questions received responses; therefore, the number responding to that particular question is indicated for each question in the text and tables. Respondents practicing in each state in the US participated, with the largest percentages coming from California (341, 16%), Texas (142, 6.7%), Florida (113, 5.3%), New York (96, 4.5%), and Colorado (92, 4.3%). The number of respondents who work in a state in which recreational marijuana was legal at the time of the survey (AK, CA, CO, DC, ME, MA, NV, OR, VM, WA) was 759 (35.6%) leaving 1,371 respondents (64.4%) working in states that had not legalized recreational marijuana as of May, 2018. Respondents were asked to indicate the year in which they graduated veterinary school. The graduation years were classified into four cohorts: 1989 or earlier (448, 21.1%), 1990–1999 (473, 22.3%), 2000–2009 (606, 28.6%), and 2010 or later (595, 28.0%).

### Knowledge Questions

Respondents were asked to indicate their knowledge level, using a 4 point Likert scale from 1 = “have no idea” to 4 = “know a lot,” in response to questions about marijuana and/or CBD products. The first question enquired about their knowledge level regarding the differences between marijuana and CBD products (*n* = 2,108). The largest number (1,207, 57.3%) reported “know some” followed by “know a lot” (426, 20.2%). When asked about the toxic effects of marijuana in dogs (*n* = 2,123), the majority reported “knowing some” (1,147, 54.0%), followed by “know a lot” (824, 38.8%). Respondents were less knowledgeable about the therapeutic effects of CBD products in dogs (*n* = 2,126); 930 (43.7%) reported “knowing some” and 745 (35.0%) reported “not knowing much.” Similarly, they were less knowledgeable about the toxic effects of CBD products in dogs (*n* = 2,126), in which 637 (30.0%) reported “knowing some” and 930 (43.7%) reported “not knowing much.” (Table 1).

**Table 1:** Veterinarians self-reported knowledge level regarding marijuana and hemp/CBD products in dogs.

	**Have no idea**	**Do not know much**	**Know some**	**Know a lot**
The differences between marijuana products and hemp/CBD products (*n* = 2108)	105 (5.0%)	370 (17.6%)	1207 (57.3%)	426 (20.2%)
The toxic effects of marijuana in dogs (*n* = 2123)	28 (1.3%)	124 (5.8%)	1147 (54.0%)	824 (38.8%)
The therapeutic effects of hemp/CBD products in dogs (*n* = 2126)	261 (12.3%)	745 (35.0%)	930 (43.7%)	190 (8.9%)
The toxic effects of hemp/CBD products in dogs (*n* = 2126)	394 (18.5%)	930 (43.7%)	637 (30.0%)	165 (7.8%)

*Data presented as number of responses and percent of total responses in parenthesis*.

**Figure 1:**
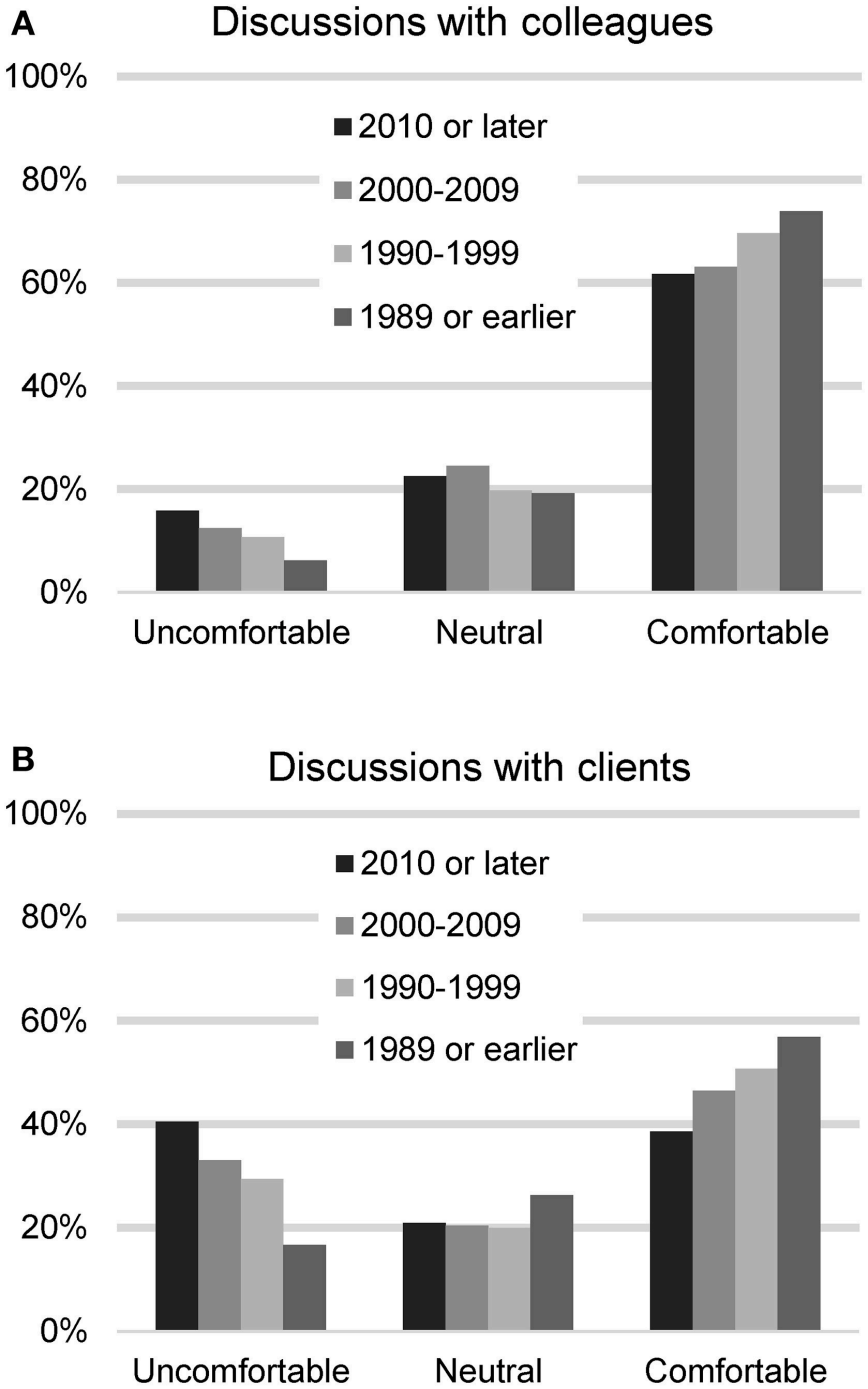
Participants' reported level of comfort in discussing CBD/hemp with colleagues **(A)** and with clients **(B)**, based on year of graduation from veterinary school.

The respondents were asked next how comfortable they feel talking to veterinary colleagues about CBD treatment for dogs (*n* = 2,127). Most felt comfortable (1,309, 61.5%), with 231 (10.9%) reporting feeling uncomfortable, 432 (20.3%) neutral, and 155 (7.3%) indicating they have not encountered the situation. When asked about their comfort level talking with clients, they were less comfortable: 967 (45.4%) reported feeling comfortable and 641 (29.9%) felt uncomfortable, 443 (20.8%) neutral, and 85 (4.0%) indicated they have not encountered the situation. A chi square test was used to assess differences in comfort level based on graduation year and legal status of recreational marijuana in the respondents' state of residence. For these analyses, those who had not encountered the situation were removed. No differences were found based on legal status of marijuana in state of practice, but differences were found based on graduation date. Recent veterinary graduates were less comfortable talking to colleagues (chi square 29.71, *p* < 0.001) as well as clients (chi square 69.22, *p* < 0.001) (Figure 1).

### Frequency of CBD-Related Consultations

Veterinarians (*n* = 2,112) were asked how often their clients enquired about CBD products and the most common response was rarely (616, 29.2%), followed by weekly (609, 28.8%), monthly (558, 26.4%), never (172, 8.1%), and daily (157, 7.4%). These responses were significantly different based on respondents' states' marijuana laws (Table 2). Clients visiting veterinarians who work in states that have legalized recreational marijuana were more likely ask about CBD for their pets (chi square 358.90, *p* < 0.001).

**Table 2:** Reported frequency of clients seeking information about CBD for pets, based on legal status of recreational marijuana in state of practice.

**State laws**	**Never**	**Rarely**	**Monthly**	**Weekly**	**Daily**
Legal (*n* = 752)	16 (2.1%)	100 (13.3%)	177 (23.5%)	353 (46.9%)	106 (14.1%)
Illegal (*n* = 1360)	156 (11.5%)	516 (37.9%)	381 (28.0%)	256 (18.8%)	51 (3.8%)

*Data presented as number of responses and percent of total responses in parenthesis*.

Participants (*n* = 2,128) were also asked to quantify how often they initiate discussions with clients about CBD products. The majority reported never (1,398, 65.7%), followed by rarely (413, 19.4%), weekly (140, 6.6%), monthly (132, 6.2%), and daily (45, 2.1%).

### Conditions for Which CBD Was Discussed

Respondents who reported client-initiated conversations about CBD products (*n* = 1,940) were next asked to identify the specific conditions or diseases for which clients were seeking information. More than one response was possible, and the most common topics were pain management, anxiety, seizures, and storm/fireworks phobias. Respondents (*n* = 730) who reported initiating conversations with clients about CBD products were also asked to identify the specific conditions or diseases for which CBD products were discussed. Multiple selections were possible, and the most commonly discussed topics were pain management, anxiety, seizures, and storm/fireworks phobias (Table 3).

**Table 3:** Common diseases/conditions for which clients sought information and for which veterinarians initiated conversations about CBD.

**Condition**	**Client sought information (*n* = 1940)**	**Veterinarian initiated conversations (*n* = 730)**
Pain management	1806 (93.1%)	614 (84.1%)
Anxiety	1341 (69.1%)	388 (53.2%)
Seizures	1089 (56.1%)	313 (42.9%)
Storm or fireworks phobias	531 (27.4%)	141 (19.3%)
Gastrointestinal diseases	203 (10.5%)	62 (8.5%)
Neoplastic/cancer	198 (10.2%)	67 (9.2%)
Motion sickness	149 (7.7%)	44 (6.0%)
Atopy or other skin conditions	132 (6.8%)	22 (3.0%)
Endocrinopathies	78 (4.0%)	15 (2.1%)
Infections	57 (2.9%)	8 (1.1%)
Appetite stimulation/anorexia	23 (1.2%)	23 (3.2%)
Palliative care	14 (0.7%)	17 (2.3%)
Osteoarthritis	12 (0.6%)	0
Other (e.g., everything, general)	67 (3.5%)	22 (3.0%)

*Data presented as number of responses and percent of total responses in parenthesis*.

### Client Communication Regarding CBD

**Figure 2:**
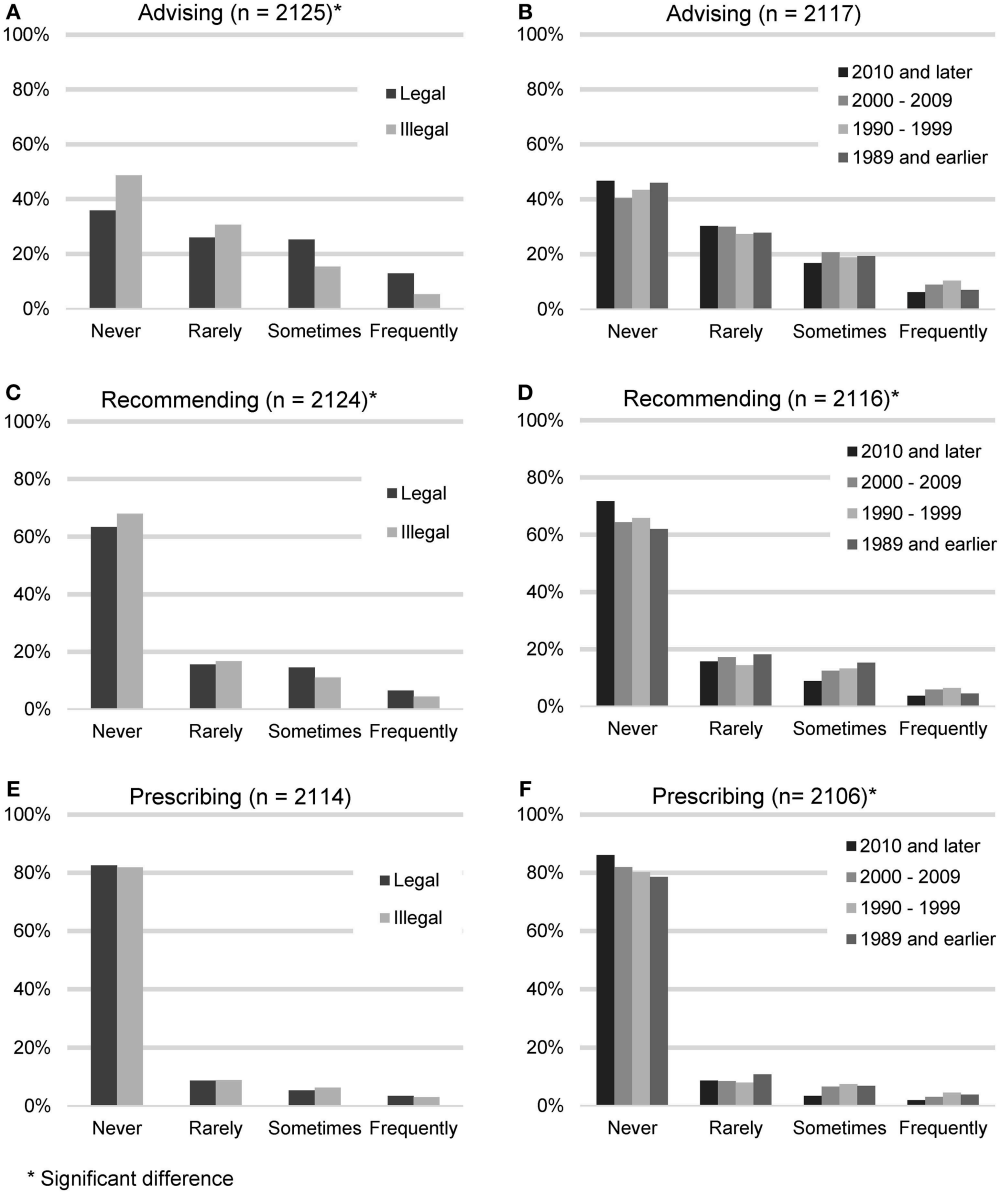
Reported frequency of advising about, recommending, and prescribing CBD products, based on the legal status of recreational marijuana in participants' state of practice **(A,C,E)** and participants' year of graduation **(B,D,F)**.

In order to gauge the degree with which veterinarians endorse the use of CBD products, participants were asked to quantify the frequency with which they advise clients about CBD products, recommend CBD products, or prescribe CBD products. These results were then analyzed based on the legal status of recreational marijuana in respondents' state of practice (Figure 2).

Advising clients about CBD products (*n* = 2,125) was considered the lowest level of endorsement. The largest number of participants reported never (938, 44.1%) or rarely (615, 28.9%) advising their clients about CBD products. A smaller number reported sometimes (401, 18.9%), or frequently (171, 8.0%). When asked for all the reasons why they did not advise clients about CBD products (*n* = 938), the most common answer was that they don't feel knowledgeable enough (639, 68.1%), followed by the field needs more research (560, 59.57%), it is illegal (458, 48.8%), concerns about toxicity (185, 19.7%) and do not think clients would be receptive (35, 3.7%). “Other” reasons included concerns about product consistency and purity or the fact that they had not been asked.

Recommending CBD products constituted the next level of endorsement. Participants were asked how often they recommend CBD products (*n* = 2,124). The majority reported never (1,409, 66.3%) or rarely (346, 16.3%). A minority reported sometimes (260, 12.2%), or frequently (109, 5.1%). When asked for all the reasons why they did not recommend CBD products (*n* = 1,409), the most common answer was that the field needs more research (912, 64.7%), followed by not feeling knowledgeable enough (888, 63.0%), it is illegal (751, 53.3%), concerns about toxicity (301, 21.4%) and do not think clients would be receptive (45, 3.2%). The most common “Other” reasons included concerns about product consistency and purity and the feeling that other options with better research exist.

Lastly, participating veterinarians were asked how often they prescribe CBD products (*n* = 2,130). The majority reported never (1,735, 82.1%) or rarely (187, 8.8%). A minority reported sometimes (125, 5.9%), or frequently (67, 3.2%). When asked for all the reasons why they did not prescribe CBD products (*n* = 1,735), the most common answer was that it is illegal (1,003, 57.8%), followed by the field needs more research (997, 57.5%), don't feel knowledgeable enough (967, 55.7%), concerns about toxicity (325, 18.7%), and do not think clients would be receptive (49, 2.8%). “Other” reasons included the fact that it can be bought over the counter and the lack of product consistency and purity.

Participants who reported living in states with legalized recreational marijuana were more likely to advise clients about CBD products (chi square 81.64, *p* < 0.001), and recommend CBD products (chi square 11.04, *p* < 0.012), but were not statistically more likely to prescribe CBD products (chi square 1.07, *p* = 0.784). Veterinarians in earlier graduating classes were more likely to recommend CBD products (chi square 20.58, *p* < 0.015), and prescribe CBD products (chi square 20.24, *p* = 0.016), but not to advise clients about CBD products (chi square 13.75, *p* = 0.132).

### Clinical Experience With CBD Products

Participants were asked if they have had any clinical experience with CBD products in dogs. This could include direct observation or client reports (*n* = 2,130). Slightly more than half reported yes (1,194, 56.1%) and 936 (43.9%) said no. Participants who indicated they had clinical experience with CBD were asked a series of questions related to their experience with specific forms of CBD as well as perceived benefits and side effects. The forms of CBD that participants were asked about included biscuits or edibles, tablets or capsules, CBD oil or extracts or tinctures, and oil or cream for topical application. Among these, participants reported the most familiarity with liquid (oil extracts or tinctures) and edible (biscuits/edibles) formulations of CBD (Table 4).

**Table 4:** Veterinarians' clinical experience with CBD products in dog.

**CBD formulation**	**None**	**A little**	**A moderate amount**	**Quite a lot**	**Extensive**
Biscuits or edibles (*n* = 1143)	465 (40.7%)	475 (41.6%)	146 (12.8%)	45 (3.9%)	12 (1.0%)
Tablets or capsules (*n* = 1141)	699 (61.3%)	305 (26.7%)	99 (8.7%)	27 (2.4%)	11 (1.0%)
Oil or extracts or tinctures (*n* = 1117)	155 (13.2%)	613 (52.1%)	266 (22.6%)	103 (8.8%)	40 (3.4%)
Oil or cream for topical application (*n* = 1140)	798 (70.0%)	253 (22.2%)	61 (5.4%)	18 (1.6%)	10 (0.9%)

*Data presented as number of responses and percent of total responses in parenthesis*.

Several potential uses of CBD products were listed and participants were asked to indicate if, in their observations or client reports, CBD products had had a harmful effect, no effect or positive/helpful on each of them. Those who responded NA (not observed/not applicable) were removed from analysis. The areas in which veterinarians reported observing (either first-hand or via client reports) the most positive effects included: analgesia for chronic and acute pain, anxiety, and seizure frequency or severity (Table 5).

**Table 5:** Perceived impact of CBD products for common canine medical conditions, listed alphabetically.

**Condition**	**Very helpful**	**Somewhat helpful**	**No effect**	**Somewhat harmful**	**Very harmful**
Analgesia for acute pain (*n* = 708)	161 (22.7%)	424 (59.9%)	116 (16.4%)	5 (0.7%)	2 (0.3%)
Analgesia for chronic pain (*n* = 1019)	348 (34.2%)	575 (56.4%)	85 (8.3%)	9 (0.9%)	2 (0.2%)
Anxiety (*n* = 833)	180 (21.6%)	546 (65.5%)	97 (11.6%)	7 (0.8%)	3 (0.4%)
Atopy (*n* = 167)	10 (6.0%)	50 (29.9%)	99 (59.3%)	4 (2.4%)	4 (2.4%)
Bacterial or fungal infection (*n* = 129)	4 (3.1%)	12 (9.3%)	107 (82.9%)	4 (3.1%)	2 (1.6%)
Diabetes mellitus (*n* = 104)	3 (2.9%)	13 (12.5%)	82 (78.8%)	3 (2.9%)	3 (2.9%)
Diarrhea (*n* = 171)	11 (6.4%)	35 (20.5%)	109 (63.7%)	12 (7.0%)	4 (2.3%)
Hyperadrenocorticism (*n* = 96)	2 (2.1%)	16 (16.7%)	73 (76.0%)	2 (2.1%)	3 (3.1%)
Hypothyroidism (*n* = 94)	4 (4.3%)	9 (9.6%)	76 (80.9%)	2 (2.1%)	3 (3.2%)
Motion sickness (*n* = 224)	31 (13.8%)	143 (63.8%)	46 (20.5%)	1 (0.4%)	3 (1.3%)
Seizure frequency or severity (*n* = 612)	132 (21.6%)	340 (55.6%)	125 (20.4%)	8 (1.3%)	7 (1.1%)
Storm or fireworks phobia (*n* = 379)	46 (12.1%)	232 (61.2%)	91 (24.0%)	8 (2.1%)	2 (0.5%)
Vomiting (*n* = 266)	32 (12.0%)	119 (44.7%)	104 (39.1%)	9 (3.4%)	2 (0.8%)

*Data presented as number of responses and percent of total responses in parenthesis*.

Participants were also asked about witnessed or reported side effects, with the most common side effect being sedation. This was reported by 28.9% of participants to occur in 1–10% of dogs. The percent of participants who reported sedation as a side effect in 11–25% of dogs was 12.5%. The next most common side effect was polyphagia, reported by 10.0% of participants to occur in 1–10% of dogs. With the exception of sedation, all other potential side effects were reported by over 80% of participants as never occurring (Table 6).

**Table 6:** Perceived side effects of CBD products for common canine medical conditions.

**Side effect**	**Never**	**1–10% of dogs**	**11–25% of dogs**	**26–50% of dogs**	**51–75% of dogs**	**76–100% of dogs**
Anorexia (*n* = 1144)	1053 (92.0%)	70 (6.1%)	11 (1.0%)	4 (0.3%)	3 (0.3%)	3 (0.3%)
Bradycardia (*n* = 1138)	1003 (88.1%)	81 (7.1%)	30 (2.6%)	16 (1.4%)	5 (0.4%)	3 (0.3%)
Constipation (*n* = 1140)	1109 (97.3%)	23 (2.0%)	2 (0.2%)	6 (0.5%)	0	0
Diarrhea (*n* = 1134)	1039 (91.6%)	76 (6.7%)	9 (0.8%)	6 (0.5%)	2 (0.2%)	2 (0.2%)
Hypertension (*n* = 1136)	1123 (98.9%)	7 (0.6%)	2 (0.2%)	3 (0.3%)	1 (0.1%)	0
Hypotension (*n* = 1136)	1078 (94.9%)	39 (3.4%)	12 (1.1%)	4 (0.4%)	2 (0.2%)	1 (0.1%)
Increased anxiety (*n* = 1139)	993 (87.2%)	87 (7.6%)	25 (2.2%)	20 (1.8%)	7 (0.6%)	7 (0.6%)
Polydipsia (*n* = 1137)	1051 (92.4%)	57 (5.0%)	17 (1.5%)	6 (0.5%)	3 (0.3%)	3 (0.3%)
Polyphagia (*n* = 1143)	949 (83.0%)	114 (10.0%)	37 (3.2%)	24 (2.1%)	15 (1.3%)	4 (0.3%)
Sedation (*n* = 1148)	560 (48.8%)	332 (28.9%)	144 (12.5%)	68 (5.9%)	25 (2.2%)	19 (1.7%)
Seizures (*n* = 1142)	1110 (97.2%)	24 (2.1%)	3 (0.3%)	2 (0.2%)	2 (0.2%)	1 (0.1%)
Tachycardia (*n* = 1137)	1066 (93.8%)	44 (3.9%)	17 (1.5%)	10 (0.9%)	0	0
Vomiting (*n* = 1139)	1035 (90.9%)	74 (6.5%)	18 (1.6%)	6 (0.5%)	4 (0.4%)	2 (0.2%)

*Data presented as number of responses and percent of total responses in parenthesis*.

### Legal/Ethical Issues and Research Regarding CBD/Marijuana

The last series of questions asked participants about their views on a variety of topics related to CBD and marijuana. Two of these questions referred to guidance on the topic offered through state organizations. For both veterinary state organizations and state veterinary boards, few participants reported feeling that these entities provided sufficient guidance regarding the use of CBD/marijuana in animals for them to practice within the state or federal law. These questions pertaining to veterinary state organizations and state veterinary boards were analyzed to determine if there were any significant differences in responses based on year of graduation or the legal status of recreational marijuana in the state in which respondents' practice veterinary medicine. A significant difference based on year of graduation was found for participants' views of the guidance offered by their veterinary state organization (chi square 30.18, *p* = 0.011), whereby those who graduated more recently report higher agreement levels. Similarly, those practicing in states with legal recreational marijuana reported higher agreement with the statement that their veterinary state organization provides sufficient guidance (chi square 11.16, *p* = 0.0480). When asked about their state veterinary board guidance, there was a difference in perception based on year of graduation, with more recent graduates reporting higher agreement levels (chi square 30.04, *p* = 0.012). No differences were found based on legal status of marijuana in state of practice (Table 7).

**Table 7:** Perception of state organizations' provision of sufficient guidance regarding the use of CBD/marijuana in animals to practice within the state or federal laws.

**State organization support**	**Strongly disagree**	**Disagree**	**Neutral**	**Agree**	**Strongly agree**	**Does not apply/don't know**
My veterinary state organization has provided sufficient guidance for me to practice within the state or federal laws (*n* = 1194)	266 (22.3%)	376 (31.5%)	286 (24.0%)	206 (17.3%)	49 (4.1%)	11 (0.9%)
My state veterinary board has provided sufficient guidance for me to practice within the state or federal laws (*n* = 1193)	262 (22.0%)	385 (32.3%)	283 (23.7%)	205 (17.2%)	50 (4.2%)	8 (0.7%)

*Data presented as number of responses and percent of total responses in parenthesis*.

The next set of questions included two questions about the perceived need for additional research, and six questions assessing views of legal status of CBD and marijuana for humans and animals. The results of these questions are summarized in Tables 8, 9. Differences in responses based on the legal status of recreational marijuana in the participants' state as well as date of graduation were assessed with chi square tests and significant differences noted. When asked about the need for additional research about the therapeutic use and toxicity of hemp/CBD in dogs, whose who graduated more recently (chi square 46.61, *p* < 0.001) as well as those practicing in states with legal marijuana (chi square 28.43, *p* < 0.001) were more likely to agree that more research is needed. There were no differences between groups for the question related to additional research on the toxicity of marijuana in dogs.

**Table 8:** Participants' views regarding the need for hemp/CBD/marijuana research.

**Research**	**Strongly disagree**	**Disagree**	**Neutral**	**Agree**	**Strongly agree**	**Does not apply/don't know**
The therapeutic use and toxicity of hemp/CBD in dogs warrants rigorous veterinary research (*n* = 1193)	34 (2.8%)	42 (3.5%)	83 (7.0%)	365 (30.6%)	666 (55.8%)	3 (0.3%)
The toxicity of marijuana in dogs warrants rigorous veterinary research (*n* = 1192)	27 (2.3%)	102 (8.6%)	209 (17.5%)	453 (38.0%)	398 (33.4%)	3 (0.3%)

*Data presented as number of responses and percent of total responses in parenthesis*.

**Table 9:** Participants' views regarding legal status of hemp/CBD/marijuana as Schedule 1 drugs.

**Legal status**	**Strongly disagree**	**Disagree**	**Neutral**	**Agree**	**Strongly agree**	**Does not apply/don't know**
CBD should remain a Schedule I drug as defined by the DEA (*n* = 1191)	706 (59.3%)	270 (22.7%)	132 (11.1%)	36 (3.0%)	45 (3.8%)	2 (0.2%)
Marijuana should remain a Schedule I drug as defined by the DEA (*n* = 1193)	534 (44.8%)	305 (25.6%)	169 (14.2%)	107 (9.0%)	78 (6.5%)	0

*Data presented as number of responses and percent of total responses in parenthesis*.

When asked if CBD should remain a Schedule I drug as defined by the DEA, those who graduated more recently report lower agreement levels (chi square 31.26, *p* = 0.008) as did those in states that had legalized marijuana (chi square 25.47, *p* < 0.001). This same pattern was observed for the question on whether marijuana should remain a Schedule I drug as defined by the DEA (graduation year: chi square 47.21, *p* < 0.001; legalized marijuana status: chi square 27.12, *p* < 0.001) (Tables 8, 9).

Participants were asked to indicate their agreement level with several statements regarding the legal status of hemp/CBD and marijuana for both animals and humans. For each statement in Table 10, there was a significant difference in stated level of agreement based on graduation year and their state's recreational marijuana laws, with the exception of hemp/CBD products for animals (only significantly different based on state's recreational marijuana laws and not graduation year) (Table 10).

**Table 10:** Participants' views regarding legal status of hemp/CBD/marijuana in animals and humans.

**Legal status**	**Strongly disagree**	**Disagree**	**Neutral**	**Agree**	**Strongly agree**	**Does not apply/don't know**	**State legal status**	**Graduation year**
I think marijuana products for humans should remain illegal at the Federal level (*n* = 1193)	599 (50.2%)	328 (27.5%)	138 (11.6%)	67 (5.6%)	37 (3.1%)	24 (2.0%)	chi square 17.99 *p* < 0.003	chi square 46.64 *p* < 0.001
I think hemp/CBD products for humans should remain illegal at the Federal level (*n* = 1194)	740 (62.0%)	309 (25.9%)	79 (6.6%)	29 (2.4%)	11 (0.9%)	26 (2.2%)	chi square 20.08 *p* = 0.001	chi square 38.17 *p* = 0.001
I think marijuana products for animals should remain illegal at the Federal level (*n* = 1,193)	474 (39.7%)	243 (20.4%)	156 (13.1%)	184 (15.4%)	92 (7.7%)	44 (3.7%)	chi square 12.79, *p* = 0.025	chi square 28.45 *p* = 0.019
I think hemp/CBD products for animals should remain illegal at the Federal level (*n* = 1,194)	697 (58.4%)	306 (25.6%)	98 (8.2%)	44 (3.7%)	14 (1.2%)	35 (2.9%)	chi square 24.86 *p* < 0.001	ns^*^

*Data presented as number of responses and percent of total responses in parenthesis*.

* *ns = not significant*.

Lastly, participants were asked to report their views on the potential benefits of marijuana and CBD products for humans as well as their support in using CBD products for animals from both a medical and ethical viewpoint. Most participants agreed or strongly agreed that both marijuana and CBD products offer benefits for humans and expressed support for use of CBD products for animals. There was a significant difference based on graduation year, with more recent graduates reporting higher agreement levels for the question related to beneficial medical uses of marijuana products for humans (chi square 25.95, *p* = 0.039). This difference was not observed for the question on the beneficial medical uses of hemp/CBD products for humans. There were also no differences based on year of graduation or laws regarding recreational marijuana in participants' state of practice, for questions related to the benefits of marijuana or CBD/hemp products for animals (Tables 11, 12).

**Table 11:** Participants' views regarding the potential medical benefits of hemp/CBD/marijuana for humans.

**Benefits**	**Strongly disagree**	**Disagree**	**Neutral**	**Agree**	**Strongly agree**	**Does not apply/don't know**
I think there are beneficial medical uses of marijuana products for humans (*n* = 1191)	36 (3.0%)	13 (1.1%)	98 (8.2%)	380 (31.9%)	634 (53.2%)	0
I think there are beneficial medical uses of hemp/CBD products for humans (*n* = 1190)	26 (2.2%)	10 (0.8%)	85 (7.1%)	358 (30.1%)	666 (56.0%)	0

*Data presented as number of responses and percent of total responses in parenthesis*.

**Table 12:** Participants' reported level of support regarding the potential medical benefits of hemp/CBD/marijuana for dogs.

**Benefits**	**Strongly disapprove**	**Disapprove**	**Neutral**	**Approve**	**Strongly Approve**
Medicinal uses of hemp/CBD products for dogs from a medical standpoint (*n* = 1193)	23 (1.9%)	32 (2.7%)	157 (13.2%)	444 (37.2%)	537 (45.0%)
Medicinal uses of hemp/CBD products for dogs from a moral standpoint (*n* = 1192)	19 (1.6%)	33 (2.8%)	248 (20.8%)	409 (34.3%)	483 (40.5%)

*Data presented as number of responses and percent of total responses in parenthesis*.

## Discussion

The current study investigated veterinarians' views and experiences surrounding CBD products for dogs. A recent national study assessing dog owners' views and behaviors surrounding CBD product usage for their dogs found that the most commonly reported use by owners of CBD products was for pain relief, followed by reduction of inflammation, and relief from anxiety (15). Pain relief was also the predominant use reported by owners in a 2016 study (16). Significant side effects were reported by <5% of owners, with most participants reported not observing any side effects (15). The significant side effect observed most frequently was lethargy yet even this effect was reported by only 3.9% of owners.

These findings assessing owners' experiences were validated in the current study. When veterinarians were asked what specific conditions or diseases clients enquired about treating with CBD products, the most common responses were pain management, anxiety and seizures. These were also the top three topics listed by veterinarians when asked for conditions about which they initiated CBD conversations with their clients.

When asked about potential benefits of CBD products for a variety of conditions, veterinarians reported observing (either first-hand or through owner reports) that CBD was the most helpful for chronic pain (reported as very helpful by 34% and somewhat helpful by 56% of veterinarians) followed by acute pain (very helpful, 23% and somewhat helpful by 60% of veterinarians). CBD was also deemed to be helpful for reducing anxiety and seizure frequency/severity by over 75% of participants. The recent clinical trials on CBD for seizures (13) and pain management (14) support these veterinarians' reported experiences.

A variety of CBD products are currently available for purchase and participants reported the most familiarity with biscuits/edibles, yet even for these, approximately 40% of veterinarians reported having no experience. Interestingly, when owners were asked what form of CBD they gave to their pets, the most common response was capsules/pills and biscuits/edibles were a distant second (56.9% compared to 29.3%).

In general, veterinarians appear reticent to initiate conversations with clients about CBD, with 85% reporting they rarely or never initiated such conversations. Few reported advising clients about CBD (73% either never or rarely), and even fewer recommended (83% either never or rarely) or prescribed (91% either never or rarely) CBD products. The most common reason given for not advising about or recommending CBD was not feeling knowledgeable enough. When asked why they did not prescribe CBD products, the most common response was the fact that it is illegal. It is interesting to note that participants who work in states that have legalized recreational marijuana are more likely to advise about and recommend CBD products, but even they do not prescribe. More experienced veterinarians were more likely to recommend and prescribe, but not to advise clients about CBD products. Yet, even with these differences, most veterinarians in the current study, do not advise about, recommend or prescribe CBD products.

Given the dearth of information available about CBD products, it is not surprising that veterinarians do not feel knowledgeable about the topic. To this point, a significant number of participants reported not knowing much or anything about the therapeutic (47%) or toxic (62%) effects of CBD products. It is also clear that the participating veterinarians do not feel they are obtaining the information they need from their state veterinary organizations or state veterinary boards. When asked, <25% of respondents feel these entities provide sufficient guidance for them to practice within the state or federal law. Unfortunately, this lack of knowledge, and therefore veterinarians' confidence to initiate CBD related conversations with their clients leaves pet owners with limited options to obtain reliable information. It is alarming, but not surprising, that CBD company websites are the source most consulted by pet owners for CBD information (16). This does not appear to be due to owners' comfort levels; over 83% of surveyed owners reported feeling comfortable talking to their vet about CBD (15). Yet, results from this current study show that only 45% of veterinarians feel comfortable talking to clients about CBD. Even more telling, only 62% of surveyed veterinarians feel comfortable talking to other veterinarians about the topic.

Veterinarians in the current sample overwhelmingly support further research into both the therapeutic use and toxicity of CBD as well as the toxicity of marijuana. The majority do not feel that CBD or marijuana should remain defined as Schedule I drugs by the DEA, nor feel that these substances should remain illegal for use in animals or humans. Taken together, these responses suggest that the veterinary community is receptive to exploring the potential of cannabis products and hungers for scientific data and clinical trials. These results are similar to those of a recent study exploring attitudes toward marijuana among medical students attending an allopathic medical school in Colorado. These students supported marijuana legal reform (reclassifying marijuana so that it is no longer a Schedule 1 substance), increased research, and medicinal uses of marijuana, but voiced concerns about potential risks and therefore, many expressed reluctance about recommending marijuana to patients (17). Another study of health care providers working in Washington, USA had similar results whereby they reported the need for additional training and education; and given their current knowledge level, did not feel comfortable recommending medical cannabis (18). New York physicians (19) as well as a national sample of oncologists (20) share similar sentiments. In fact, these challenges are faced by physicians worldwide (21–23). A limitation of this study is that only veterinarians who subscribe to VIN (Veterinary Information Network) participated in this study. Although VIN has a large member base, it does not represent all veterinarians. It is possible that members of VIN may have different views on this topic than all veterinarians; therefore, we must be cautious to not extrapolate these results to the entire profession. Nevertheless, the authors believe the results are informative on this timely topic and the conclusion that more research is needed on the potential benefits and potential toxicities of CBD products can be generalized to the profession outside of VIN. The authors believe that VIN membership is reflective of the overall population of veterinarians in the U.S. VIN consists of 34, 917 members located in all 50 states. The average age of VIN members is 45.5 years compared to the average age of veterinarians in the U.S. of 44.1 years. Women constitute 69% of VIN members and 65% of U.S. veterinarians (24).

The sales of natural pet supplements nearly doubled between 2008 and 2014 with no signs of slowing down; U.S. retail sales are projected to grow 3–5% annually (25). The use of CBD products for animals is expected to increase as pet owners look for alternative ways to care for their pets. And while pet treats and food are regulated, pet supplements fall in a gray unregulated zone because they are not classified as drugs or food. Given the constantly changing laws and regulations on cannabis products as well as the lack of scientific study, obtaining accurate information on cannabis products is critically important. Certainly, current laws and political forces make it challenging for veterinarians to gain the information they need to feel confident discussing CBD with their clients and offering sound advice, yet it is imperative for the veterinary field to rise to this challenge. Given the positive feelings expressed by veterinarians in this study, it is suggested that all those affected by both the potential benefits as well as the risks, work together for legislative change that would allow for the expansion of knowledge needed to best capitalize on this potential medical tool for companion animals.

## Author Contributions

All authors listed have made a substantial, direct and intellectual contribution to the work, and approved it for publication.

### Conflict of Interest Statement

The authors declare that the research was conducted in the absence of any commercial or financial relationships that could be construed as a potential conflict of interest.
